# Bivalirudin for Recurrent Pediatric Venous Thromboembolism: A Case Report of Heparin Resistance and Thrombectomy

**DOI:** 10.14740/jmc5306

**Published:** 2026-04-29

**Authors:** Sarah Shelley, Leslie Matthews Richards

**Affiliations:** a Heritage College of Osteopathic Medicine – Dublin Campus, Dublin, Ohio and Ohio University, Athens, OH, USA; b Department of Anesthesiology & Pain Medicine, Nationwide Children’s Hospital, Columbus, OH, USA

**Keywords:** Pediatric venous thromboembolism, Deep vein thrombosis, Pulmonary embolism, Heparin resistance, Bivalirudin, Mechanical thrombectomy, Direct thrombin inhibitor, Anticoagulation failure

## Abstract

Venous thromboembolism (VTE) is rare in pediatric patients, and spontaneous thrombosis in otherwise healthy children presents unique diagnostic and therapeutic challenges. We describe a 15-year-old male who presented with abrupt right leg pain and swelling. Imaging revealed extensive deep venous thrombosis (DVT) involving the right iliac, femoral, and popliteal systems, along with bilateral pulmonary emboli (PEs). He was started on an unfractionated heparin (UFH) infusion but therapeutic anticoagulation was never achieved despite escalating doses, consistent with heparin resistance. During hospitalization, the patient developed worsening clot burden and limb swelling. His hospital course was complicated by worsening clot burden requiring two mechanical thrombectomies. Given failure of UFH, anticoagulation was transitioned to bivalirudin, which stabilized his clinical course and improved perfusion and clot burden on follow-up imaging. He was discharged on enoxaparin, though repeat evaluation at 3 months showed recurrent thrombosis and persistent pulmonary emboli. He was subsequently transitioned to oral dabigatran, after which follow-up ultrasound demonstrated improvement. This case highlights the importance of recognizing heparin resistance in pediatric patients with persistently subtherapeutic anticoagulation, considering mechanical thrombectomy for extensive or limb-threatening thrombosis, and using direct thrombin inhibitors such as bivalirudin when UFH fails. Although experience outside extracorporeal support is limited, bivalirudin may be a safe and effective alternative for pediatric VTE management. This case demonstrates successful use of bivalirudin after heparin resistance and thrombectomy in a pediatric patient without cardiac disease or extracorporeal support, highlighting a novel application of direct thrombin inhibition and the need for further studies to define standardized pediatric dosing, monitoring, and safety outcomes.

## Introduction

Venous thromboembolism (VTE) includes blood clots formed in the deep venous system as well as those that may embolize to the lungs. Deep venous thromboses (DVTs) most commonly involve the lower extremities and typically present with swelling or pitting edema, redness, tenderness, and superficial venous distension. Clinical manifestations of PE include sudden onset of dyspnea or worsening of preexisting respiratory symptoms, chest pain, syncope or dizziness due to hypotension or shock, hemoptysis, tachycardia, or tachypnea [1–3]. The global burden of VTE is significant, with approximately 10 million cases annually, though the incidence is markedly higher in adults than in children [2, 3]. Pediatric VTE is far less common, with estimated incidence rates ranging from 0.07 to 0.14 per 10,000 children in the general population and up to 58 per 10,000 among hospitalized children [2, 4, 5]. The presence of a central venous access devices represents the most significant risk factor in children. In contrast, spontaneous thrombosis in previously healthy children is rare and poses complex diagnostic and management challenges due to limited clinical experience and the absence of standardized protocols [5].

Anticoagulation is the first-line treatment for symptomatic VTE management in pediatric patients, aimed at preventing clot extension, embolization, and recurrence. Initial therapy most often involves either low molecular weight heparin (LMWH) or unfractionated heparin (UFH), depending on the clinical setting. LMWH is typically preferred in outpatient care for its ease of administration and predictable pharmacokinetics, while UFH is commonly used in the inpatient setting due to its rapid onset of action, reversibility, and ability to be titrated through monitoring of activated partial thromboplastin time (aPTT) or anti-Xa levels [6–8]. Heparin resistance, a rare clinical scenario in which therapeutic aPTT or anti-Xa levels fail to be reached despite escalating doses, may be attributed to antithrombin deficiency or increased heparin clearance and often necessitates further investigation or changes in therapy [8].

VTE recurrence while on therapeutic anticoagulation is uncommon in the pediatric patient but may occur in the setting of large clot burden, underlying thrombophilia, or anticoagulation failure [8]. In such cases, direct thrombin inhibitors like bivalirudin may be considered. Bivalirudin has shown utility in pediatric patients undergoing extracorporeal membrane oxygenation (ECMO) or cardiac surgery, but its use in pediatric VTE management outside of these settings is not well established [8–10]. This case highlights a unique presentation of extensive VTE in a previously healthy pediatric patient who demonstrated heparin resistance, with recurrent thrombosis despite escalating doses of UFH, necessitating a transition to bivalirudin for intraoperative and post-procedural anticoagulation following a second thrombectomy.

## Case Report

### Investigations

Review of this case and presentation in this format followed the guidelines of the Institutional Review Board of Nationwide Children’s Hospital. A 15-year-old, 49.3 kg male with past medical history only significant for constipation and intermittent hematochezia presented to the emergency room with abrupt onset of right leg pain and swelling. He had no history of recent surgery, travel, trauma, and denied fever, chest pain, nausea, vomiting, or abdominal pain. Family history was negative for clotting disorders. The patient reported being generally sedentary but had no history of prolonged immobilization.

### Diagnosis

**Figure 1:**
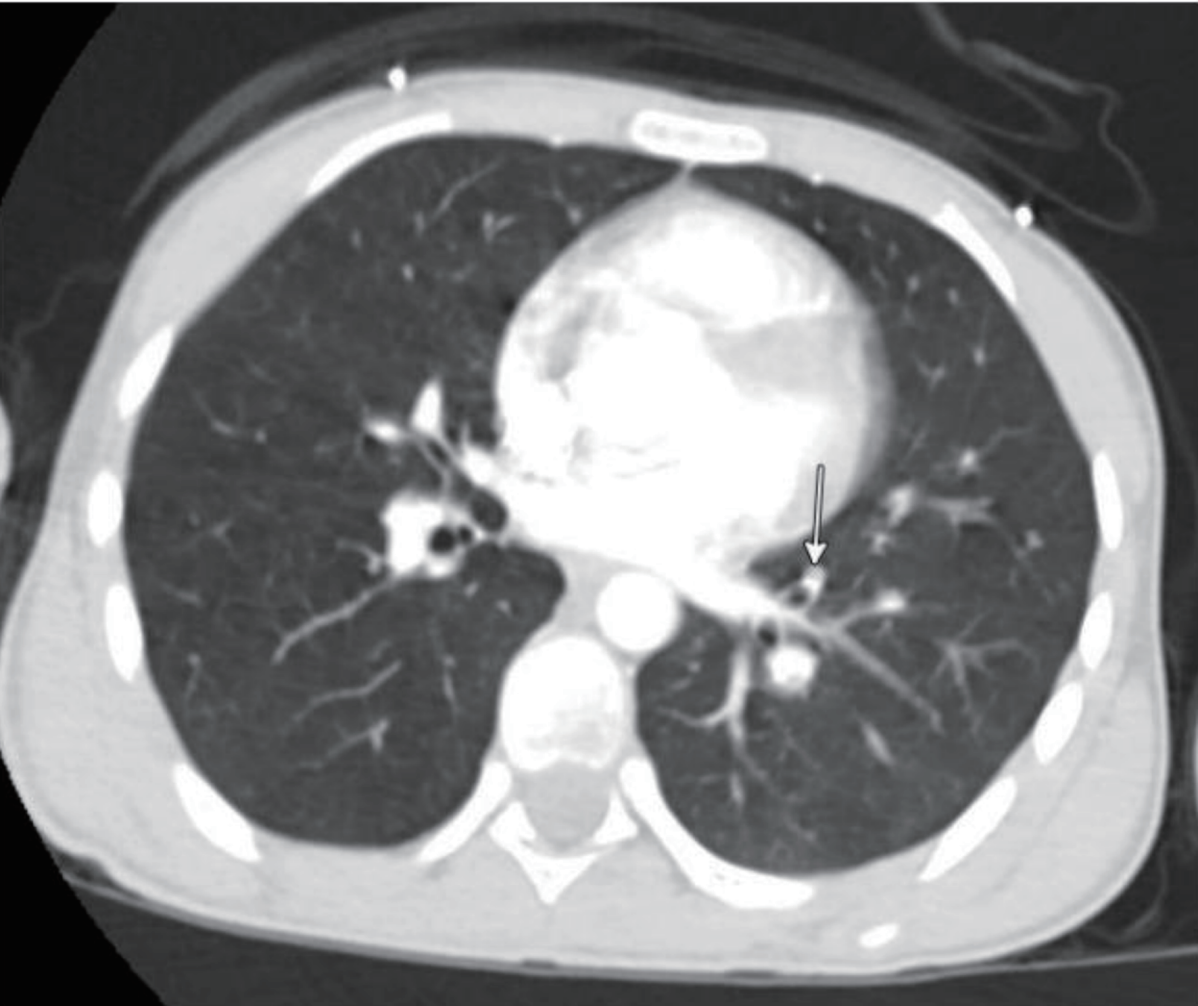
Computed tomography of the chest. Arrow points to one of many pulmonary emboli.

At an outside hospital, computed topography (CT) angiography revealed right lower extremity DVT extending from the femoral vein to the foot, along with bilateral lower lobe pulmonary emboli (PEs) (Fig. 1). Laboratory studies were notable for leukocytosis (white blood cell (WBC) 23), significant anemia (hemoglobin 5.3 g/dL), and thrombocytosis (platelets 700). He received one unit of packed red blood cells (pRBCs) prior to transfer and a second unit upon arrival at Nationwide Children’s Hospital. Initial vital signs were temperature 99.2 °F (37.3 °C), pulse 123 bpm, respirations 24, blood pressure 125/87 mm Hg, and oxygen saturation 100% on 2 L of O_2_ for comfort. On physical exam, he was ill-appearing and tachycardic with dry mucous membranes, pale skin, and delayed capillary refill. Though not in respiratory distress, he was tachypneic with diminished breath sounds at the bases. His right leg was diffusely swollen, plethoric, and extremely tender with intact distal pulses. An electrocardiogram (ECG) showed sinus tachycardia (heart rate (HR) 118), left axis deviation, T-wave inversion in V1, and right bundle branch block. There were no prior ECGs for comparison. B-type natriuretic peptide and troponin levels were normal. An echocardiogram showed no evidence of right heart strain.

### Treatment

The patient was started on an UFH infusion at 20 units/kg/h with a target anti-Xa level of 0.25–0.70 units/mL. He was admitted to the pediatric intensive care unit due to his clinical presentation and extensive clot burden. During pediatric intensive care unit (PICU) admission, the patient had an episode of frank hematuria while on a subtherapeutic heparin infusion (anti-Xa < 0.35), prompting a temporary reduction in therapeutic targets to prophylactic anti-Xa levels (0.15–0.30). Despite heparin boluses and dose titrations, therapeutic anti-Xa levels were unreached. The patient’s hemoglobin dropped from 8.3 to 6.9 g/dL, raising concern for bleeding versus hemolysis. An inferior vena cava (IVC) filter was placed on hospital day 2 due to risk of embolization and concern for anticoagulation failure.

Additional CT angiography revealed continued extensive thrombosis of the right common, internal, and external iliac veins extending into the lower extremity veins. Associated findings included marked edema and inflammation of the right leg musculature and gluteal muscles. The first mechanical thrombectomy was performed on hospital day 3, using an Inari device via right popliteal and right internal jugular access. A large clot burden was removed and the IVC filter was retrieved without complication. The patient returned to the PICU extubated and in stable condition, and anticoagulation was continued with a heparin infusion.

Despite repeated boluses and infusion rate increases (up to 43 units/kg/h) of UFH, anti-Xa levels and aPTTs remained below target. On hospital day 5, the patient had a venous ultrasound of the right lower extremity, revealing complete reocclusion of the external iliac vein, great saphenous vein, common femoral vein, superficial femoral vein, and nearly complete occlusion of the popliteal vein and posterior tibial and peroneal veins. Clinical exam revealed worsening swelling of the right thigh, scrotum, and flank, with numbness and increasing pain in the right foot. Given ongoing clot progression and laboratory evidence of persistent subtherapeutic anticoagulation, the patient was transitioned to a continuous infusion of bivalirudin at 0.125 mg/kg/h.

**Figure 2:**
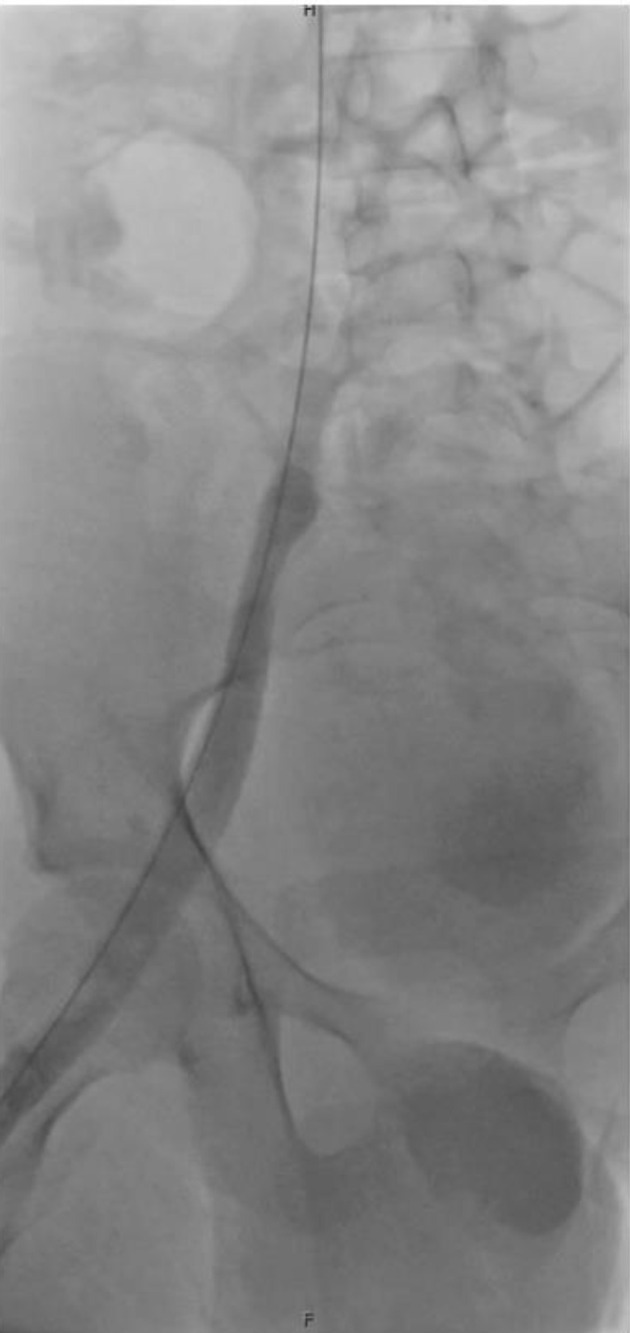
Fluoroscopic image from interventional radiology showing thrombotic occlusion of the internal iliac vein.

On hospital day 6, the patient underwent a second thrombectomy in interventional radiology (Fig. 2). Intraoperatively, a continuous bivalirudin infusion was infused at 0.5 mg/kg/h and titrated sequentially to 0.6 mg/kg/h, 0.75 mg/kg/h, and finally reduced to 0.15 mg/kg/h at the completion of the procedure. Intraoperative monitoring of activated clotting time (ACT) was used to guide dosing adjustments. Pharmacochemical and suction thrombectomy was performed via right internal jugular and posterior tibial vein access. A large volume of acute thrombus was removed with restoration of in-line flow. Both internal iliac systems were noted to be occluded, but no IVC thrombus was seen.

**Figure 3:**
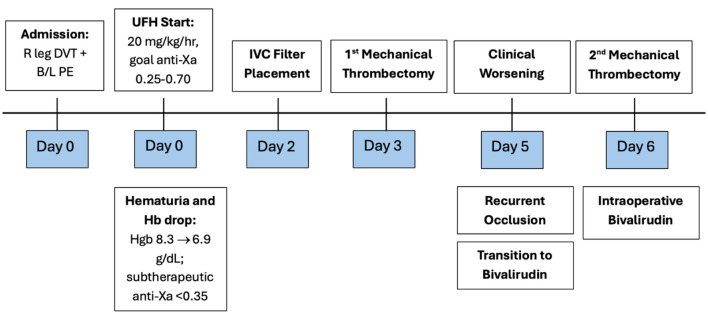
Timeline depicting the hospital course. Key events include initiation of UFH with subtherapeutic anticoagulation, clinical worsening with increased clot burden, two mechanical thrombectomies, and transition to bivalirudin.

Postoperatively, the patient remained on a continuous infusion of bivalirudin at 0.19 mg/kg/h with an aPTT goal of 60–80 s. He demonstrated gradual clinical improvement with reduction in swelling and improved perfusion. Repeat ultrasound on hospital day 10 showed improved thrombus burden. Bivalirudin infusion was continued until hospital day 14, when he was transitioned to enoxaparin 60 mg twice daily. He was discharged home on day 16 with improved mobility, family instruction on enoxaparin administration, and plans for outpatient anti-Xa monitoring (Fig. 3).

### Follow-up and outcomes

At his 6-week follow-up, vascular ultrasound demonstrated continued improvement in clot burden, but a 3-month follow-up showed reaccumulation of clot and worsening clot burden. Three-month follow-up chest CT showed persistent pulmonary emboli. Due to presumed enoxaparin failure, he was transitioned to oral dabigatran, a direct thrombin inhibitor. Initial ultrasound following this change showed reduction in clot burden and improvement in flow.

## Discussion

This case highlights the diagnostic and therapeutic challenges of managing massive VTE in a pediatric patient who experienced thrombus recurrence while receiving UFH. In our patient, UFH failed to achieve therapeutic anticoagulation despite escalating doses and close anti-Xa monitoring, consistent with clinical heparin resistance. Heparin resistance is defined as an inability to achieve therapeutic anticoagulation goals despite increasing heparin infusion rates. It reflects a shift in the heparin dose-response curve and often requires doses exceeding 35,000 units per day in adults or > 70 units/kg/h in children to achieve target aPTT or anti-Xa levels [11, 12]. Antithrombin is a necessary cofactor for heparin’s therapeutic activity, and inadequate levels are thought to play a role in heparin resistance [11]. Although relatively rare in pediatrics, heparin resistance has been documented and is a critical consideration in the context of therapeutic failure. When heparin resistance is suspected or confirmed, a direct thrombin inhibitor (DTI), such as bivalirudin, provides a valuable alternative [12].

In pediatric VTE, mechanical thrombectomy becomes imperative in high-risk scenarios. These include massive or limb-threatening thrombosis, anticoagulation failure, extensive central clot burden, or severe tissue compromise [5]. In our patient, recurrent thrombosis despite heparin and signs of impending limb threat (marked swelling, scrotal edema, flank discomfort, and reduced distal sensation) prompted urgent intervention.

**Figure 4:**
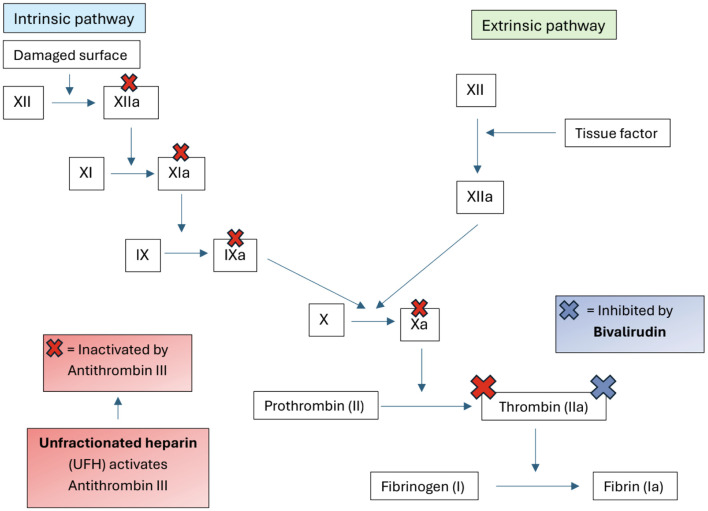
Schematic comparison of anticoagulant mechanisms for UFH and bivalirudin. UFH activates antithrombin III to inhibit factors XIIa, XIa, IXa, Xa, and thrombin (IIa), while bivalirudin directly inhibits thrombin independent of antithrombin III.

Bivalirudin is a synthetic, parenteral, bivalent DTI that binds both the active catalytic site and the anion-binding exosite of thrombin [13]. Unlike heparin, it does not require antithrombin for activity and inhibits both free and clot bound thrombin (Fig. 4). These pharmacologic properties make it especially useful in patients with antithrombin deficiency or ongoing thrombus formation despite heparin therapy [12]. Bivalirudin was initially approved for anticoagulation in adults with unstable angina undergoing percutaneous transluminal coronary angioplasty and later for use in percutaneous coronary intervention with glycoprotein IIb/IIIa inhibitors. Over time, its off-label use has expanded, including applications in patients with heparin-induced thrombocytopenia (HIT), extracorporeal life support (ECLS), and vascular assist devices. In pediatric populations, it has shown promise in scenarios of heparin failure, HIT, continued thrombosis despite therapeutic heparin, and during cardiac surgery or ECMO. The most significant disadvantage of bivalirudin is the lack of prospective randomized studies demonstrating the efficacy and safety profile in pediatric patients [12–14].

Bivalirudin exhibits linear pharmacokinetics with a short elimination half-life of approximately 25 min in adults and minimal plasma protein binding aside from thrombin. It is primarily cleared through proteolytic cleavage by thrombin (80%) with the remaining fraction (20%) excreted unchanged by the kidneys. In pediatric patients, pharmacokinetic profiles are age-dependent: neonates demonstrate higher clearance rates and lower plasma concentrations, while older children exhibit slower clearance and higher exposure. Despite this variability, the elimination half-life remains relatively consistent across age groups, ranging from 25 to 28 min. These age-dependent differences underscore the need for individualized dosing regimens and close therapeutic monitoring, particularly in the context of renal dysfunction [12, 14].

While bivalirudin is generally well-tolerated, its side effect profile warrants careful monitoring, especially in pediatric patients where experience remains limited. In adults, major bleeding rates associated with bivalirudin range from 2.3% to 3.7% with minor bleeding occurring in up to 13.6% of patients. Other common adverse effects include hypotension (12%), hypertension (6%), nausea (15%), vomiting (6%), bradycardia (5%), and injection site pain (8%). Although pediatric specific data are more limited, initial studies suggest a comparable safety profile. Of the children, 1.8% experienced major bleeding, while 11% experienced minor bleeding, most commonly small hematomas or catheter-site oozing. Additionally, thrombosis was reported in 7.3% patients, including one case that required thrombolytic intervention [12]. Despite the absence of a specific reversal agent, bivalirudin’s short half-life and predictable pharmacokinetics allow for rapid cessation of anticoagulation effects simply by stopping the infusion. However, in cases of overdose or bleeding, hemodialysis or hemofiltration may be employed to facilitate drug removal [14].

Currently, there is no universally established dosing range for bivalirudin in infants and children. However, based on available studies and clinical experience, an effective regimen for thromboprophylaxis or treatment includes a bolus of 0.125 to 0.25 mg/kg, followed by a continuous infusion of 0.125 to 0.25 mg/kg/h. For procedures requiring deeper anticoagulation, such as cardiac catheterization or cardiopulmonary bypass (CPB), larger bolus doses (0.5 to 1 mg/kg) and infusion rates up to 2.5 mg/kg/h have been employed. Some centers additionally administer 50 mg of bivalirudin directly into the CPB circuit to ensure adequate anticoagulation [12].

Monitoring anticoagulation with bivalirudin is commonly achieved via aPTT in non-procedural settings and ACT intraoperatively due to frequency of monitoring and faster turnaround time. aPTT is checked every 2 to 4 h with a therapeutic target of 1.5 to 2.5 times baseline. While ACT and aPTT correlate with bivalirudin concentration at lower levels, their accuracy diminishes at higher doses or during procedures like CPB. Newer assays, including ecarin chromogenic assay (ECA) and dilute thrombin time (dTT), may provide more reliable correlation but are not widely available or standardized in pediatric care [12, 14].

In this case, bivalirudin was initiated after failure to achieve therapeutic anticoagulation with UFH, enabling effective perioperative management during repeat thrombectomy and subsequent clinical improvement. This report expands the limited pediatric literature on bivalirudin use for heparin-resistant VTE outside extracorporeal support and underscores the need for prospective studies to define standardized dosing, monitoring strategies, and safety outcomes in children.

### Learning points

This case highlights several critical lessons in pediatric VTE management. First, heparin resistance should be suspected when therapeutic anticoagulation targets are not achieved despite escalating UFH doses, as failure to recognize it may lead to recurrent thrombosis and clinical deterioration. Second, mechanical thrombectomy can be lifesaving in massive or limb-threatening thrombosis, particularly when anticoagulation is inadequate or clot burden is extensive. Third, bivalirudin offers a safe and effective alternative anticoagulant in pediatric patients with heparin resistance or anticoagulation failure. Finally, long-term monitoring and follow-up imaging are essential, as recurrent thrombosis may occur despite procedural intervention and anticoagulation, highlighting the need for individualized therapy and consideration of alternative agents when conventional therapy fails.

## Data Availability

Any inquiries regarding supporting data availability of this study should be directed to the corresponding author.

## Abbreviations

ACT: activated clotting time
aPTT: activated partial thromboplastin time
CPB: cardiopulmonary bypass
CT: computed topography
ECA: Ecarin chromogenic assay
ECMO: extracorporeal membrane oxygenation
DTI: direct thrombin inhibitor
dTT: dilute thrombin time
DVT: deep venous thrombosis
ECG: electrocardiogram
ECLS: extracorporeal life support
HIT: heparin-induced thrombocytopenia
IVC: inferior vena cava
LMWH: low molecular weight heparin
PE: pulmonary emboli
PICU: pediatric intensive care unit
pRBCs: packed red blood cells
UFH: unfractionated heparin
VTE: venous thromboembolism
